# Developmental dynamics of voltage-gated sodium channel isoform expression in the human and mouse brain

**DOI:** 10.1186/s13073-021-00949-0

**Published:** 2021-08-23

**Authors:** Lindsay Liang, Siavash Fazel Darbandi, Sirisha Pochareddy, Forrest O. Gulden, Michael C. Gilson, Brooke K. Sheppard, Atehsa Sahagun, Joon-Yong An, Donna M. Werling, John L. R. Rubenstein, Nenad Sestan, Kevin J. Bender, Stephan J. Sanders

**Affiliations:** 1 Department of Psychiatry and Behavioral Sciences, UCSF Weill Institute for Neurosciences, University of California, San Francisco, San Francisco, CA 94158 USA; 2 Department of Neuroscience and Kavli Institute for Neuroscience, Yale School of Medicine, New Haven, CT 06510 USA; 3 Department of Neurology, UCSF Weill Institute for Neurosciences, University of California, San Francisco, San Francisco, CA 94158 USA; 4 School of Biosystem and Biomedical Science, College of Health Science, Korea University, Seoul, 02841 Republic of Korea; 5 Laboratory of Genetics, University of Wisconsin-Madison, Madison, WI 53706 USA; 6 Program in Cellular Neuroscience, Neurodegeneration, and Repair and Yale Child Study Center, Yale School of Medicine, New Haven, CT 06510 USA; 7 Department of Psychiatry, Yale University School of Medicine, New Haven, CT 06520 USA; 8 Department of Genetics, Yale University School of Medicine, New Haven, CT 06520 USA; 9 Department of Comparative Medicine, Program in Integrative Cell Signaling and Neurobiology of Metabolism, Yale School of Medicine, New Haven, CT 06510 USA; 10 Institute for Human Genetics, University of California, San Francisco, San Francisco, CA 94158 USA; 11 Bakar Computational Health Sciences Institute, University of California, San Francisco, San Francisco, CA 94158 USA

**Keywords:** Isoform, Splicing, Voltage-gated sodium channel, Autism spectrum disorder, Intellectual disability, Developmental delay, Epileptic encephalopathy, Seizures, Exon 5A, Exon 5N

## Abstract

**Background:**

Genetic variants in the voltage-gated sodium channels *SCN1A*, *SCN2A*, *SCN3A*, and *SCN8A* are leading causes of epilepsy, developmental delay, and autism spectrum disorder. The mRNA splicing patterns of all four genes vary across development in the rodent brain, including mutually exclusive copies of the fifth protein-coding exon detected in the neonate (5N) and adult (5A). A second pair of mutually exclusive exons is reported in *SCN8A* only (18N and 18A). We aimed to quantify the expression of individual exons in the developing human brain.

**Methods:**

RNA-seq data from 783 human brain samples across development were analyzed to estimate exon-level expression. Developmental changes in exon utilization were validated by assessing intron splicing. Exon expression was also estimated in RNA-seq data from 58 developing mouse neocortical samples.

**Results:**

In the mature human neocortex, exon 5A is consistently expressed at least 4-fold higher than exon 5N in all four genes. For *SCN2A*, *SCN3A*, and *SCN8A*, a brain-wide synchronized 5N to 5A transition occurs between 24 post-conceptual weeks (2nd trimester) and 6 years of age. In mice, the equivalent 5N to 5A transition begins at or before embryonic day 15.5. In *SCN8A*, over 90% of transcripts in the mature human cortex include exon 18A. Early in fetal development, most transcripts include 18N or skip both 18N and 18A, with a transition to 18A inclusion occurring from 13 post-conceptual weeks to 6 months of age. No other protein-coding exons showed comparably dynamic developmental trajectories.

**Conclusions:**

Exon usage in *SCN1A*, *SCN2A*, *SCN3A*, and *SCN8A* changes dramatically during human brain development. These splice isoforms, which alter the biophysical properties of the encoded channels, may account for some of the observed phenotypic differences across development and between specific variants. Manipulation of the proportion of splicing isoforms at appropriate stages of development may act as a therapeutic strategy for specific mutations or even epilepsy in general.

**Supplementary Information:**

The online version contains supplementary material available at 10.1186/s13073-021-00949-0.

## Background

Genetic variation in the genes *SCN1A*, *SCN2A*, *SCN3A*, and *SCN8A* are a major cause of epileptic encephalopathy, autism spectrum disorder (ASD), and developmental delay [1–3]. These four homologous genes encode voltage-gated sodium channels (Na_V_1.1, Na_V_1.2, Na_V_1.3, and Na_V_1.6, respectively) that are critical for a range of functions in the central nervous system [4], including axonal action potential initiation and propagation [5, 6], dendritic excitability [7, 8], macroscopic anatomical development [9], and activity-dependent myelination [10]. The functional role, subcellular location, expression level, and isoform selection of voltage-gated sodium channels vary across development, and understanding this relationship is critical for understanding the etiology of the associated disorders and their therapeutic management [7, 11–19]. While some isoform-level differences have been assayed in rodents and mature human brains [15, 20–23], the trajectories in the developing human cortex have not been described [24].

**Fig. 1:**
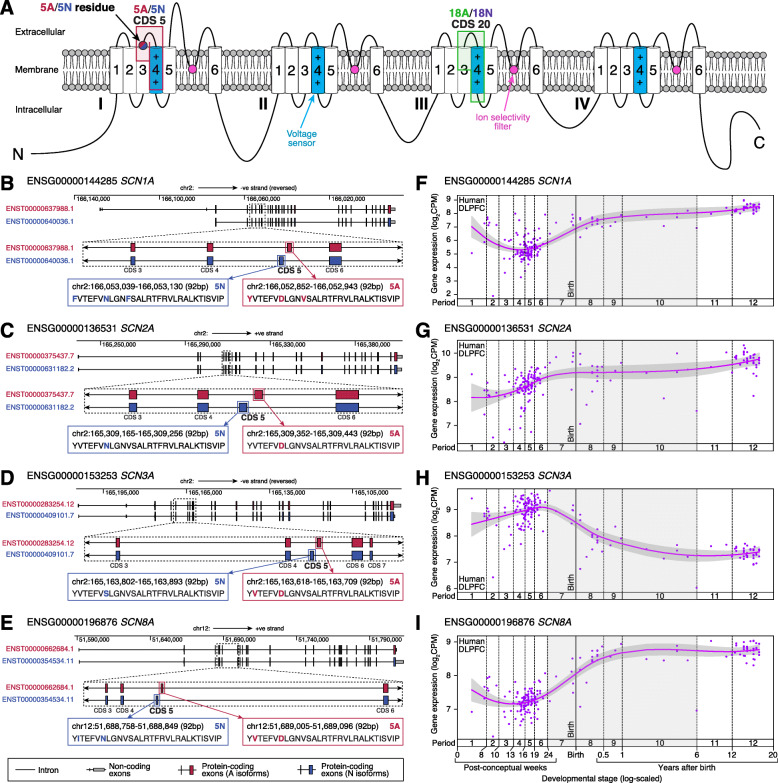
Splicing isoforms in voltage-gated sodium channels. **A** Voltage-gated sodium channels are composed of four similar domains (I, II, III, IV), each of which includes six transmembrane segments with extracellular or intracellular linkers. The fourth transmembrane segment (S4) in each domain acts as a voltage sensor. Between the fifth and sixth transmembrane segment (S5, S6) is a pore loop that forms the ion selectivity filter. The fifth protein-coding exon (5A/5N, CDS 5) encodes a portion of the first domain, while the 20th protein-coding exon (18A/18N, CDS 20) encodes a similar portion of the third domain. **B** Location, genomic coordinates (GRCh38/hg38), and amino acid sequence of the “5A” and “5N” exons in *SCN1A*. **C**–**E** The data in “B” is repeated for the genes *SCN2A*, *SCN3A*, and *SCN8A*. **F** Patterns of whole-gene expression of *SCN1A* in the human dorsolateral prefrontal cortex (DLPFC) across prenatal and postnatal development from the BrainVar dataset [33]. **G**–**I** The data in “F” is repeated for the genes *SCN2A*, *SCN3A*, and *SCN8A*. CDS: coding sequence; CPM: counts per million. Genomic coordinates are based on GRCh38/hg38 using GENCODE v31 gene definitions

Sodium channel genes are composed of multiple exons, which can be protein-coding (CDS for CoDing Sequence), untranslated regions (UTRs), or non-coding exons (NCEs). Gene isoforms are differing combinations of these exons, which can change the amino acid sequence of the encoded proteins (protein isoforms or proteoforms). The best-characterized isoform change across these four sodium channels are the two mutually exclusive copies of the fifth protein-coding exon [16, 25]. This exon encodes part of the first domain of the Na_V_ channel, including the end of transmembrane segment S3, most of transmembrane segment S4, and a short extracellular linker connecting these two segments (Fig. 1A). In humans, each copy of this fifth protein-coding exon is 92 nucleotides in length, encoding 30 amino acids, of which one to three amino acids vary between the two exon copies for each gene (Fig. 1B–E). “A” isoforms include the ancestral and canonical copy of exon 5 (5A), with an aspartic acid residue (Asp/D) encoded at position 7 of 30 [26]. “N” isoforms use the alternative copy of exon 5 (5N), with an asparagine residue (Asn/N) at position 7 of 30 in *SCN1A*, *SCN2A*, and *SCN8A* and a serine residue (Ser/S) in *SCN3A*. Despite this relatively small change in protein structure, differential inclusion of 5A or 5N can have marked effects on channel function. Indeed, these splice isoforms can alter channel electrophysiological characteristics [24, 27], the functional impacts of variants associated with seizure [24], neuronal excitability [28], response to anti-epileptics [21, 22, 29], and seizure-susceptibility [28].

The utilization of the 5A or 5N varies across development, with 5N generally being expressed at higher levels in the neonatal period while 5A predominates in adults [30]. This switch is defined best in mouse, where the 5A:5N ratio varies by gene and brain region along with developmental stage [20]. For *Scn2a* in mouse neocortex, the 5A:5N ratio is 1:2 at birth (postnatal day 0/P0) and flips to 3:1 by P15. For both *Scn3a* and *Scn8a*, 5A predominates throughout the postnatal period with a 2:1 ratio at P0 increasing to 5:1 by P15 [20]. *Scn1a* lacks a functional copy of 5N in the mouse genome. Similar developmental profiles currently have not been reported for humans beyond the 5A/5N utilization in *SCN1A* in adults, in which a 5A:5N ratio of over 5:1 was observed in the temporal cortex and hippocampus of adult surgical resections [21, 22].

In addition to the 5A/5N switch, a similar developmental shift in mutually exclusive exons has been reported for “exons 18A or 18N” in *SCN8A* only, regulated by the RNA-binding protein RBFOX1 [15, 23, 31]. Using GENCODE human v31 gene definitions [32], 18A maps to the 20th protein-coding exon of major *SCN8A* isoforms (CDS 20, Fig. 1A), while 18N encodes the 8th and last protein-coding exon (CDS 8) of a shorter transcript with eight protein-coding exons (ENST00000548086.3, Additional file 1: Fig. S1). In the embryonic mouse brain, most *SCN8A* transcripts include 18N or skip both 18N and 18A, leading to non-functional channels, while 18A predominates in the adult mouse and human brain [15, 23].

Here, we present data on the utilization of GENCODE-annotated protein-coding exons in four seizure-associated voltage-gated sodium channels in the human and mouse neocortex across development. We demonstrate a synchronized transition from 5N to 5A utilization between 24 post-conceptual weeks (2nd trimester) and six years of age across all four voltage-gated sodium channels and a transition from 18N to 18A in *SCN8A* from 13 post-conceptual weeks to 6 months of age. These isoform differences can modify the function of the encoded voltage-gated sodium channels, raising the potential that interventions, such as antisense oligonucleotides, could be used to modify the isoform ratio as a potential therapy for disorders caused by variants in sodium channel genes or epilepsy.

## Methods

### Genomic data

To quantify the relative proportion of protein-coding exon expression across development in the human cortex, we assessed bulk-tissue RNA-seq data from 176 *postmortem* neurologically normal samples of the dorsolateral prefrontal cortex (DLPFC, *N* = 167 older than 10 post-conception weeks) or frontal cerebral wall (*N* = 9 younger than 10 post-conception weeks) from the BrainVar cohort (Additional File 2: Table S1) [33]. These deidentified samples ranged from 6 post-conception weeks to 20 years of age with 104 males and 72 females. The BrainVar cohort also has corresponding whole-genome sequencing data that were used to derive per sample genotypes, as described previously [33]. To assess exon expression across brain regions, we assessed bulk-tissue RNA-seq data from 607 *postmortem* neurologically normal samples from 41 individual brains across 11 cortical regions, hippocampus, amygdala, mediodorsal nucleus of the thalamus, striatum, and cerebellar cortex from the BrainSpan cohort (Additional File 2: Table S1) [12, 34]. The deidentified BrainSpan samples ranged from 8 post-conception weeks and 40 years of age with 23 males and 18 females. To assess corresponding patterns of exon expression in mouse cortex across development, we assessed 58 cortical samples with bulk tissue RNA-seq data in wildtype C57BL/6 J (JAX: 000664) mice (Additional File 2: Table S1). Thirty-four of these were generated as controls for ongoing experiments. These animals were housed with littermates in a vivarium with a 12-h light, 12-h dark cycle. Embryonic day 0.5 was defined as noon on the day the vaginal plug was detected. Data for the remaining 24 mice were downloaded from GEO [35].

### Exon expression

**Fig. 2:**
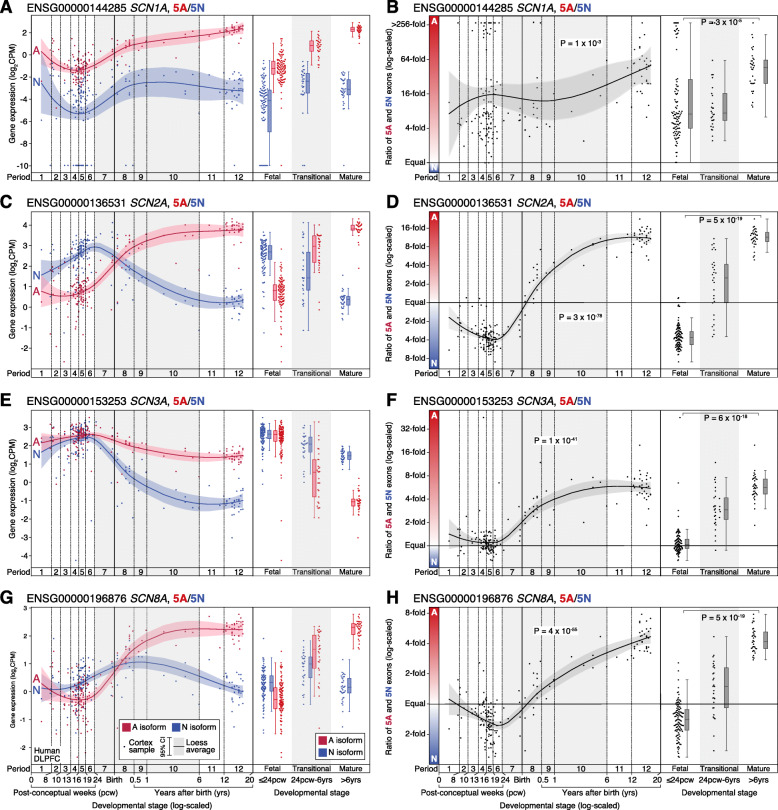
Expression of 5A and 5N in the human cortex across development. **A** The expression of 5A (red) and 5N (blue) in *SCN1A* is shown for 176 BrainVar human cortex (DLPFC) samples across development (points). On the left, the colored line shows the Loess smoothed average and 95% confidence interval (shaded region). On the right, boxplots show the median and interquartile range for the same data, binned into fetal, transitional, and mature developmental stages. **B** The ratio of 5A and 5N expression from panel **A** is shown across development (left) and in three developmental stages (right). **C**–**H** Panels **A** and **B** are repeated for the genes *SCN2A*, *SCN3A*, *SCN8A*. For comparison, the 5A/5N ratio is shown on the same *y*-axis in Additional file 1: Fig. S3, and equivalent plots for CDS four and six are shown in Additional file 1: Fig. S4. CPM: Counts per million; DLPFC: Dorsolateral prefrontal cortex. Statistical tests: **B**, **D**, **F**, **H** Left panel, linear regression of log_2_(5A:5N ratio) and log_2_(post-conceptual days). Right panel, two-tailed Wilcoxon test of log_2_(5A:5N ratio) values between fetal and mature groups

To assess exon expression in the human cortex, the 100 bp paired-read RNA-seq data from BrainVar were aligned to the GRCh38.p12 human genome using STAR aligner [36], and exon-level read counts for GENCODE v31 human gene definitions were calculated with DEXSeq [37] and normalized to counts per million (CPM) [38]. Despite the similar amino acid sequence, the nucleotide sequence of 5A and 5N is sufficiently differentiated across the four genes that 100 bp reads align unambiguously to one location in the genome [39]. Reads were detected in 5A and 5N for all samples, across all four genes, with the exception of *SCN1A* for which 31 of 176 samples (17.6%) had no detectable 5N reads (Fig. 2A). Along with quantifying the expression of 5N and 5A (Fig. 2), we also assessed expression for the surrounding constitutive exons, as a control. The BrainSpan data were analyzed using the same methods. For the mouse cortical data, the same analysis methods were used but with alternative references, specifically the GRCm38/mm10 genome and GENCODE vM25 gene definitions. A similar approach was used to assess the utilization of 18A and 18N in *SCN8A*/*Scn8a*.

### Intron splicing

**Fig. 3:**
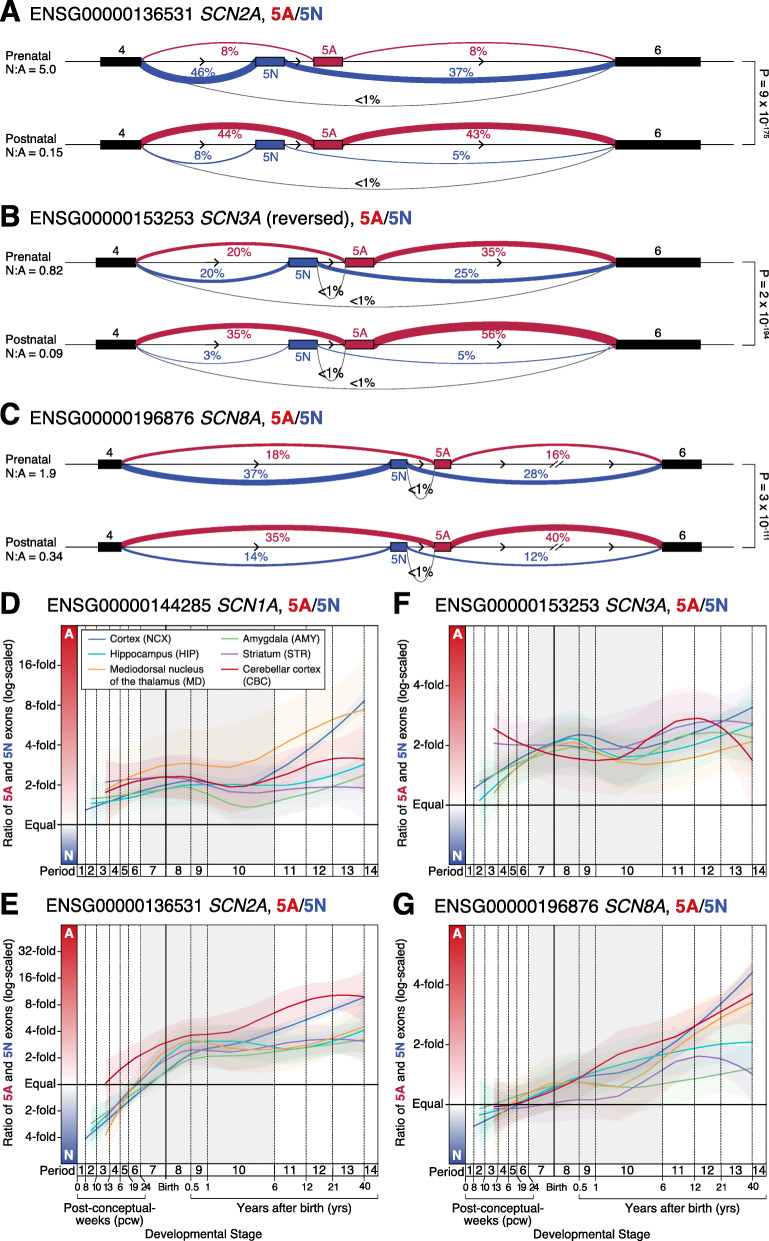
Orthogonal analysis of voltage-gated sodium channel gene splicing in the developing human brain. **A** Sashimi plot of splicing in prenatal (top, *N* = 112 samples) and postnatal (bottom, *N* = 60 samples) DLPFC for *SCN2A*. Linewidth reflects the proportion of split reads observed for each intron compared to all split reads between CDS 4 and CDS 6, this value is also shown as a percentage. Introns related to 5A inclusion are shown in red, those related to 5N inclusion are shown in blue, and others are in grey. **B**, **C** Equivalent plots for *SCN3A* (a negative-strand gene with the orientation reversed to facilitate comparison to the other two genes) and *SCN8A*. **D** The ratio of 5A and 5N expression is shown across development for *SCN1A* in six human brain regions. For each region, the colored line shows the Loess smoothed average and 95% confidence interval (shaded region). Equivalent data across 11 cortical regions are shown in Additional file 1: Fig. S6. **E**–**G** This analysis is repeated for *SCN2A*, *SCN3A*, and *SCN8A.* Statistical tests: **A**–**C** *P* values compare the prenatal and postnatal cluster using a Dirichlet-multinomial generalized linear model, as implemented in Leafcutter [41]

We applied a complementary approach to detecting 5A and 5N exon usage by assessing intron splicing via reads that map across exon-exon junctions in the same BrainVar samples. Reads were aligned with OLego aligner [40] using the same genome build and gene definitions as for exon expression. Clusters of differential intron splicing were identified with Leafcutter [41] and differences across development were detected by comparing 112 prenatal samples to 60 postnatal samples, excluding 4 samples in the transitional late fetal period [33]. No cluster was detected for 5A/5N in *SCN1A*, preventing assessment across development, but 5A/5N clusters were identified and assessed for the other three genes and for 18A/18N in *SCN8A* (Figs. 3 and 5).

### Quantitative trait locus (QTL) analysis

Common variants with a minor allele frequency ≥ 5% in both prenatal (*N* = 112) and postnatal (*N* = 60) samples and Hardy Weinberg equilibrium *p* value ≥ 1 × 10^−12^ were identified previously [33]. Variants within one million basepairs of each sodium channel gene were extracted and integrated with the Leafcutter clusters, along with the first five principal components calculated from common variants identified in whole-genome sequencing data from these samples and 3804 parents from the Simons Simplex Collection [33, 42] to predict splicing QTLs with FastQTL [43]. This analysis was performed on all samples, prenatal-only samples, and postnatal-only samples, with false discovery rate (FDR) estimated from the results of each analysis using the Benjamini-Hochberg procedure [44]. To assess the correlation of 5N expression for the SNP rs3812718, genotypes were extracted for chr2:166,053,034 C>T (GRCh38) and compared with 5N expression calculated by DEXSeq, as described above.

### Statistical analysis

The 5A:5N expression ratio was calculated from normalized exon expression values (CPM). Linear regression was used to assess whether this ratio varied across development by comparing the log-transformed 5A:5N ratio to log-transformed post-conceptual days (Fig. 2). The difference in 5A:5N ratio was also assessed between the mid-late fetal samples (*N* = 112) and childhood/adolescent/young adult samples (*N* = 35) with a two-tailed Wilcoxon test. To compare intron splicing between prenatal and postnatal samples, we used the p-values estimated with a Dirichlet-multinomial generalized linear model, as implemented in Leafcutter [41]. The same approach was used to calculate 18A:18N ratios for *SCN8A*.

## Results

### Expression of voltage-gated sodium channels in the human cortex

Gene expression varies dramatically across development for many genes, especially during the late-fetal transition, during which half the genes expressed in the brain undergo a concerted increase or decrease in expression [12, 33, 34, 45]. To assess gene-level developmental trajectories, we analyzed bulk-tissue RNA-seq in 176 *postmortem* samples from the BrainVar cohort (104 male, 72 female, spanning 6 post-conceptual weeks to 20 years after birth) from the dorsolateral prefrontal cortex (DLPFC, *N* = 167 older than 10 post-conception weeks) or frontal cerebral wall (*N* = 9 younger than 10 post-conception weeks) [33]. The gene-level expression profile of all four voltage-gated sodium channels changes during this late-fetal transition (Fig. 1F–I), with *SCN1A*, *SCN2A*, and *SCN8A* expression rising from mid-fetal development through infancy to early childhood, while *SCN3A* expression falls.

### Developmental trajectories of 5A and 5N expression in the human cortex

The majority of protein-coding exons follow the expression trajectory of their parent gene across development (Additional file 1: Fig. S2); however, all four sodium channels show dynamic changes in the utilization of 5A/5N (Fig. 2, Additional file 1: Fig. S3). This is especially marked for *SCN2A* and *SCN8A*, where 5N is expressed at a higher level than 5A in the mid-fetal brain but this reverses soon after birth. Plotting the 5A:5N ratio allows exon utilization to be assessed independent of changes in gene expression (Fig. 2). All four genes show changes in the 5A:5N ratio across development, with a modest change for *SCN1A* (7.1 fetal to 45.7 childhood/adolescent; *p* = 3 × 10^−5^, two-sided Wilcoxon test, Fig. 2B) and dramatic changes for *SCN2A* (0.27 to 11.4; *p* = 5 × 10^−19^, Fig. 2D), *SCN3A* (1.0 to 5.7; *p* = 6 × 10^−18^, Fig. 2F), and *SCN8A* (0.7 to 4.2; *p* = 5 × 10^−19^, Fig. 2H). As a control, we applied this approach to assess the ratio of the neighboring protein-coding exons: coding sequence (CDS) 4 and CDS 6. We observed no developmental shift in the 4:6 ratio for *SCN1A*, *SCN2A*, and *SCN3A*; however, the exon 4:6 ratio is marginally higher than expected in the prenatal period for *SCN8A* (0.82 vs. 0.66; 9 × 10^−10^, Additional file 1: Fig. S4). This developmental variation in *SCN8A* is not observed for the surrounding protein-coding exons and reflects a modest increase in CDS 4 expression in the prenatal period, based on the expected expression given the exon length (Additional file 1: Fig. S2, Additional file 1: S5).

### Intron splicing around 5A and 5N in the human cortex

To verify that mutually exclusive use of 5A and 5N underlies the observed exon expression changes (Fig. 2), we considered RNA-seq reads that spanned exon-exon junctions to quantify intron splicing in BrainVar. Clusters of differential intron splicing corresponding to 5A/5N usage were identified by Leafcutter for *SCN2A*, *SCN3A*, and *SCN8A* (Fig. 3A–C), but not *SCN1A*, likely due to the consistently low expression of N isoforms (Fig. 2). The splicing patterns for *SCN2A*, *SCN3A*, and *SCN8A* are consistent with the observed exon expression changes (Figs. 2 and 3) and at least 99% of reads are consistent with mutually exclusive 5A/5N utilization.

### Developmental trajectories of 5A and 5N expression across human brain regions

To assess 5A and 5N expression across multiple brain regions we repeated the exon expression analysis in 607 samples from 41 individual *postmortem* human brains in the BrainSpan cohort [12, 34]. A similar trajectory of 5A:5N utilization was observed for all four sodium channels across the cortex, hippocampus, amygdala, striatum, mediodorsal nucleus of the thalamus, and cerebellar cortex (Fig. 3D–G) and across eleven cortical regions (Additional file 1: Fig. S6).

### Developmental trajectories of 5A and 5N expression in the mouse cortex

**Fig. 4:**
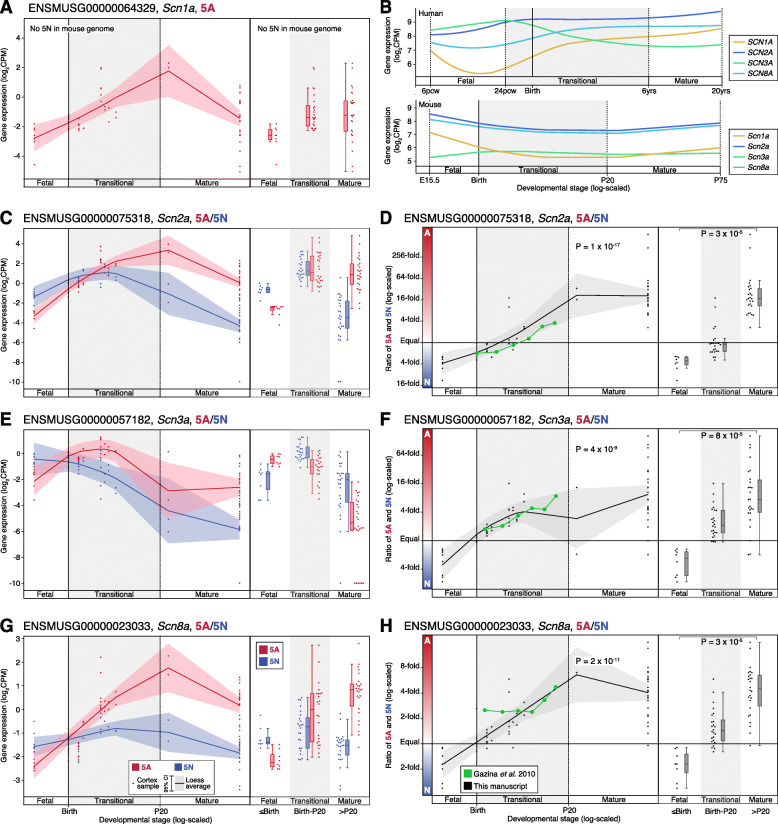
Expression of 5A and 5N in the mouse cortex across development. **A** The expression of 5A (red) in *Scn1a* is shown for 58 mouse cortex samples across development (points); no functional 5N equivalent is present in the mouse genome. On the left, the colored line shows the Loess smoothed average and 95% confidence interval (shaded region). On the right, boxplots show the median and interquartile range for the same data, binned into fetal, transitional, and mature developmental stages. **B** The Loess smoothed average expression of the four voltage-gated sodium channels in human cortex (top, Fig. 1) and mouse cortex (bottom). **C** Panel **A** is repeated for *Scn2a*, with the addition of 5N expression (blue). **D** The ratio of 5A and 5N expression from panel ‘C’ is shown across development (left) and in three developmental stages (right). Values reported previously in mouse cortex are shown on the same scale in green for comparison [20]. **E-H** Panels **C** and **D** are repeated for the genes *Scn3a*, *Scn8a*. CPM: Counts per million. Statistical tests: **D**, **F**, **H** Left panel, linear regression of log_2_(5A:5N ratio) and log_2_(post-conceptual days). Right panel, two-tailed Wilcoxon test of log_2_(5A:5N ratio) values between fetal and mature groups

We repeated the analysis of sodium channel 5A/5N expression using bulk-tissue RNA-seq data from the mouse cortex across development (*N* = 58; E15.5 to P75). Our data are consistent with the 5A:5N ratios described previously (Fig. 4), however, by assessing a wider developmental period we observe more substantial differences at the extremes of development: *Scn2a* (0.3 fetal to 17.3 mature; *p* = 3 × 10^−5^, two-sided Wilcoxon test, Fig. 4C), *Scn3a* (0.4 to 7.4; *p* = 8 × 10^−5^, Fig. 4E), and *Scn8a* (0.6 to 4.5; *p* = 3 × 10^−5^, Fig. 4G). Mice lack a functional 5N exon in *Scn1a*.

### No evidence of common polymorphisms regulating 5A or 5N utilization

A common polymorphism (rs3812718, GRCh38 chr2:166,053,034 C>T, IVS5N+5G>A) has previously been associated with epilepsy, seizures, and response to anti-epileptics [21, 22, 29, 46, 47], though this variant did not reach genome-wide significance in a mega-analysis of epilepsy [48]. Prior analyses of expression in the adult human temporal cortex showed evidence that the homozygous variant allele (TT in DNA, AA in cDNA) was associated with reduced utilization of 5N in *SCN1A* [21, 49]. We do not observe evidence for such a relationship in the prenatal or postnatal prefrontal cortex (Additional file 1: Fig. S7) and this polymorphism is not identified as a splicing quantitative trait locus (sQTL) in GTEx (50). Furthermore, this variant is not predicted to alter splicing behavior using the SpliceAI algorithm [50]. We did not identify rs3812718 as an expression quantitative trait locus (eQTL) in the BrainVar cohort and do not see evidence of a relationship to *SCN1A* gene-wide expression (Additional file 1: Fig. S7), however, in GTEx, the TT genotype is associated with increased *SCN1A* expression in the adult human basal ganglia (*p* = 1 × 10^−10^) [51].

### Developmental trajectories of 18A and 18N expression in *SCN8A*

**Fig. 5:**
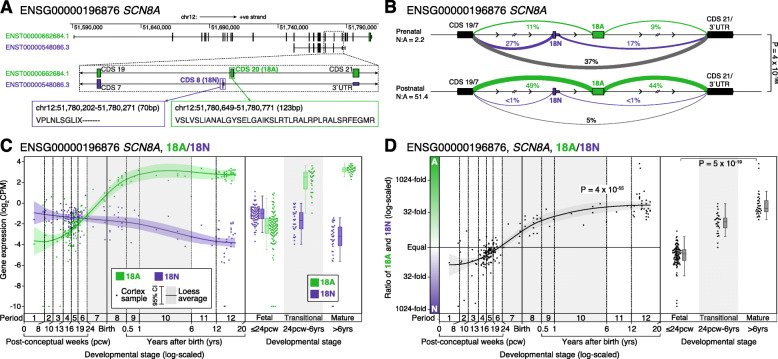
Developmental trajectories of CDS 20 (18A/18N) in human cortex in *SCN8A*. **A** Location, genomic coordinates (GRCh38/hg38), and amino acid sequence of the 18A and 18N exons in *SCN8A*. **B** Sashimi plot of intron splicing in prenatal (top, *N* = 112 samples) and postnatal (bottom, *N* = 60 samples) dorsolateral prefrontal cortex. Linewidth reflects the proportion of split reads observed for each intron compared to all split reads between the exon before and after, this value is also shown as a percentage. Introns related to 18A exon inclusion are shown in green, those related to 18N exon inclusion are shown in purple, and others are in grey. **C** Expression of the 18A (green) and 18N (purple) for 176 BrainVar human dorsolateral prefrontal cortex samples across development (points). On the left, the colored line shows the Loess smoothed average with the shaded area showing the 95% confidence interval. On the right, boxplots show the median and interquartile range for the same data, binned into fetal, transitional, and mature developmental stages. **D** The 18A:18N ratio is shown for each sample from panel **C** across development (left) and binned into three groups (right). CPM: Counts per million; Statistical analyses: **B** Dirichlet-multinomial generalized linear model, as implemented in Leafcutter [41]. **D** Left panel, linear regression of log_2_(18A:18N ratio) and log_2_(post-conceptual days). Right panel, two-tailed Wilcoxon test of log_2_(18A:18N ratio) values between fetal and mature groups

We next considered the developmental timing of the transition between 18A and 18N in *SCN8A* (Figs. 1A and 5A). Intron splicing shows a robust difference between prenatal and postnatal human dorsolateral prefrontal cortex (*P* = 4 × 10^−185^, Fig. 5B), with the prenatal period characterized by high frequencies of transcripts excluding 18A, either including 18N or skipping both 18N and 18A, while in the postnatal cortex 18A is included in 93% of reads. Considering exon expression (Fig. 5C, D), the expression of 18A increases markedly over development and this is distinct from other protein-coding exons for *SCN8A* (Additional file 1: Fig. S2). Consistent with prior analyses of the human brain [23], the 18A/18N transition begins around 13 post-conceptual weeks and continues till 6 months of age, with both timepoints being earlier than the equivalents for 5A/5N in *SCN8A* and the other genes.

### Other annotated protein-coding exons with distinct developmental trajectories

**Fig. 6:**
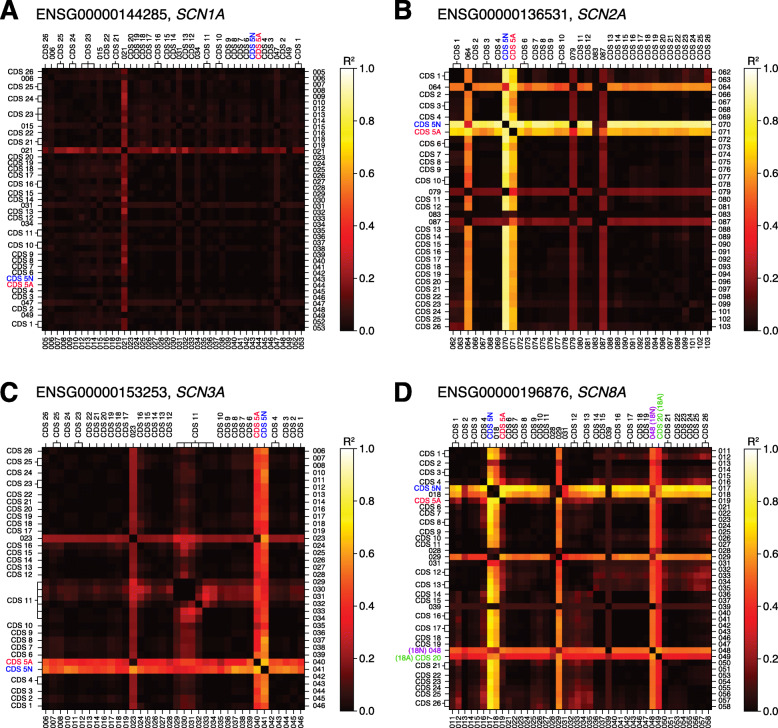
Identification of protein-coding exons with complex developmental trajectories. **A** The correlation between the ratio of CPM expression between pairs of exons (log-scaled) and developmental stage (post-conceptual days, log-scaled) for *SCN1A* was assessed with a linear model (e.g., Fig. 2B). The *R*^2^ value of each exon pair is shown as a heat map with ‘hot’ colors representing exon pairs with high *R*^2^ values for which variation in the ratio is correlated with developmental age, i.e., pairs of exons that show substantially different expression across development. Exon numbers from DEXSeq (Additional file 3: Table S2) are shown on the bottom and right and equivalent CDS numbers are shown on the top and left (see Additional file 3: Table S2). **B**–**D** The analysis is repeated for *SCN2A*, *SCN3A*, and *SCN8A*

To assess whether other protein-coding exons undergo distinct developmental transitions (Additional file 1: Fig. S2), we calculated the ratios of all pairs of protein-coding exons within each of the four sodium channel genes and assessed whether the ratio was correlated with developmental stage using linear regression. This is the same calculation used to quantify the 5A/5N and 18A/18N transitions (Figs. 2 and 5D) and distinguishes exons with expression profiles that differ from the rest of the gene (e.g., 5A in *SCN2A*), rather than simply being expressed at reduced levels, suggesting alternative regulatory processes (Additional file 1: Fig. S2). Visualizing the *R*^2^ values of these correlations provides a simple method to identify such distinct developmental trajectories (Fig. 6). Aside from 5A/5N and, in *SCN8A*, 18A/18N, no protein-coding exons common to most isoforms (consistent CDS in Additional file 1: Fig. S2) show differential expression across development, but a few weakly expressed protein-coding exons specific to a small number of isoforms do (variable CDS in Additional file 1: Fig. S2) (Fig. 6).

GENCODE defines seven variable CDS exons for *SCN1A* (DEXSeq divisions: 006, 015, 021, 031, 034, 047, 049; Additional file 3: Table S2, Fig. 6A). Of these, only 021 shows a distinct developmental trajectory (Fig. 6A), with reduced postnatal expression relative to other *SCN1A* exons (Additional file 1: Fig. S2). This result is verified by the intron splicing data (*p* = 6 × 10^−91^, Leafcutter).

In *SCN2A*, the 5A/5N trajectories stand out clearly (Fig. 6B). There are four variable CDS exons (DEXSeq divisions: 064, 079, 083, 087; Additional file 3: Table S2, Fig. 6B), three of which have distinct developmental trajectories (Fig. 6B, Additional file 1: Fig. S4): 064 (Additional file 1: Fig. S2, *P* = 2 × 10^−12^, Leafcutter), 079 (Additional file 1: Fig. S2, *P* = 7 × 10^−33^, Leafcutter), 087 (Additional file 1: Fig. S2, *P* = 2 × 10^−20^, Leafcutter). The single variable CDS exon in *SCN3A*, 023 (Additional file 3: Table S2, Fig. 6C), varies across development (Additional file 1: Fig. S2, *P* = 3 × 10^−80^, Leafcutter). Finally, aside from 18N, there are five variable CDS exons in *SCN8A* (DEXSeq divisions: 018, 028, 029, 031, 039; Additional file 3: Table S2, Fig. 6D) of which 018 and 029 vary across development (Fig. 6D), but neither of these is validated by Leafcutter.

## Discussion

Using transcriptomic data from 176 human prefrontal cortex samples and 607 samples across 16 brain regions, we characterized the developmental patterns for protein-coding exons in *SCN1A*, *SCN2A*, *SCN3A*, and *SCN8A* (Fig. 6, Additional file 1: Fig. S4). We observed a coordinated increase in the 5A:5N ratio between 24 post-conceptual weeks (2nd trimester) and six years of age across brain regions, which is synchronized with widespread transcriptomic changes in the brain during the late-fetal transition [33, 34]. This is preceded by a similar increase in the 18A:18N ratio in *SCN8A* from 13 post-conceptual weeks to 6 months of age, which is regulated by RBFOX1 [15, 23, 31]. By analyzing a wider developmental window than prior analyses [20, 21, 23, 49], we observed more dynamic changes and larger disparities in exon expression. These splicing changes modify channel function [24, 27], neuronal behavior [28], and clinical phenotypes [21, 22, 29] and interact with specific disorder-associated variants [24].

Recent advances have shown that differential splicing patterns can be effective therapeutic targets in humans, for example through intrathecal antisense oligonucleotides (ASOs) [52, 53], and rapid progress is being made with ASOs to modify gene-wide expression in rodent models of some voltage-gated sodium channel disorders [54–56]. Manipulating the expression of specific exons in these genes may represent a complementary therapeutic strategy; we consider several therapeutic scenarios.

First, for individuals with disorder-associated genetic variants within the 30 amino acids encoded by either 5A or 5N, expressing the other copy of 5A/5N could skip the variant. Theoretically, this approach could benefit individuals with both loss-of-function (protein-truncating variants, missense, splice site) and severe gain-of-function (missense) variants and at least eight cases of epileptic encephalopathy have been identified with variants in 5A of *SCN2A* or *SCN8A* [13, 19, 57, 58]. Epilepsy resulting from many of these variants is poorly managed with antiepileptic drugs [13], which either block sodium channels with limited isoform specificity or target other mechanisms (e.g., other ion channels). Whether such a strategy offers benefits over simply reducing overall channel expression [54, 56] remains to be seen, however, there may be additional effects of decreasing expression of both the normal and variant-containing alleles. Such conditions could mimic cases of *SCN2A* haploinsufficiency, associated with autism spectrum disorder and intellectual disability [13, 58, 59]. Furthermore, some children with *SCN2A* haploinsufficiency experience seizures, and recent work suggests that lowering *SCN2A* expression levels below 50% may increase the prevalence of such seizure conditions [60–62]. Thus, for this subset of patients, splice-altering ASOs may provide a wider therapeutic window than ASOs that reduce gene expression. The success of such a therapy would depend upon the proportion of transcripts expressing the alternate 5th exon and the ability of this exon to functionally replace the original 5th exon.

Second, in *SCN8A*, gain-of-function variants lead to epileptic encephalopathy. Reducing the levels of the encoded Na_V_1.6 channel should improve symptoms, as demonstrated by an ASO that degrades *SCN8A* mRNA in mice [54]. Interventions that promote exon skipping of 18A or exon switching from 18A to 18N would prevent translation of functional Na_V_1.6 channels, leading to a similar effect. All interventions aimed at reducing *SCN8A* levels would require very careful dosing since *SCN8A* haploinsufficiency is strongly associated with intellectual disability [63].

Third, splice isoforms can also affect the biophysical effects of variants outside of 5A and 5N. Two variants associated with benign infantile seizures—M252V and L1563V—exhibit biophysical changes only when expressed on 5N isoform [19, 27]. Since benign infantile seizures resolve spontaneously these are not candidates for novel, potentially risky, therapies, however, they demonstrate the existence of variants with isoform-specific impacts on the biophysical properties of the channel. Three recently characterized epileptic encephalopathy-associated variants in *SCN2A*—T236S, E999K, and S1336Y—all exhibit more pronounced alterations in their electrophysiological properties in 5N Na_V_1.2 isoforms compared to 5A isoforms [24]. For individuals with these and equivalent variants, tilting expression towards the 5A isoform could provide some symptomatic improvement, especially during infancy. Conversely, if variants exist with more pronounced effects in the 5A isoform, then encouraging 5N expression may be beneficial. Critically, such an approach relies on detailed electrophysiological characterization of specific variants, both in heterologous expression systems and in neuronal cell lines or rodent models, as data obtained for individual variants in expression systems can vary based on recording conditions and co-expression of sodium channel auxiliary subunits [24, 27]. At present, the burden of characterization limits the translational potential of such allele-specific interventions compared with gene-specific approaches [54–56]; however, technological advances or large-scale characterization efforts could provide future opportunities.

Finally, modifying 5A/5N splicing might aid seizure control in older children and adults. At this age, the 5A isoform is predominantly utilized in both *SCN2A* or *SCN8A*, which are critical for regulating the excitability of glutamatergic neurons [7, 11, 60, 61]. Reverting expression to the fetal/neonatal state by encouraging 5N utilization could reduce the excitability of cortical glutamatergic neurons, potentially limiting seizures [60]. For ASOs, the repeated intrathecal administration would limit such an approach to the most severe cases of epilepsy, however, small molecules can also modify splicing behavior [64]. It remains to be seen whether this approach could offer therapeutic benefits above and beyond existing antiepileptic drugs.

Our analysis was limited by the use of short-read transcriptomic data, leading us to focus on quantifying exon-level expression (Fig. 2) and splice junction usage (Fig. 3), rather than relying on estimates of isoform utilization (Additional file 1: Fig. S1). We also elected to focus on protein-coding transcripts and exons defined by GENCODE (v31) rather than attempting de novo transcriptome assembly. Emerging long-read transcriptomic technology may substantially expand estimates of isoform and exon diversity but these technologies have not been applied to the developing human brain at scale [65, 66]. We also note that transcriptomic data is only partially predictive of protein levels and other factors, including channel transport and degradation, may influence the impact of isoforms on neuronal function. Comparing results from the human and mouse cortex (Figs. 2 and 4), more substantial differences in gene and exon expression may be observed at earlier embryonic times in the mouse or with larger sample sizes. In addition, the use of bulk-tissue transcriptomic data limits our ability to assess how individual cell types or cell states contribute to the observed isoform trajectories. Technological and methodological advances may provide insights at cell-level resolution in the future [67].

## Conclusions

Dramatic differences in exon usage of *SCN1A*, *SCN2A*, *SCN3A*, and *SCN8A* observed in rodent brains also occur in the developing human brain, beginning in fetal development and continuing through early childhood. These changes in splicing affect the biophysical properties of the encoded channels and are likely to contribute to differences in phenotype observed between individuals with different variants and across development. Manipulation of these splicing patterns may have therapeutic applications.

## Supplementary Information

**Additional file 1:.** Supplementary Figures; Supplementary Figures 1 to 7 accompanying the main text.

**Additional file 2:.** Table S1 – Sample information; Metadata on human and mouse samples used in this study, including age, sex, tissue, and sequencing conditions.

**Additional file 3:.** Table S2 – Exon information; Details of the voltage-gated sodium channel gene exons featured in this study.

**Additional file 4:.** Supplementary materials

## Data Availability

The BrainVar data are available through the PsychENCODE Knowledge Portal: syn21557948 on Synapse.org (https://www.synapse.org/#!Synapse:syn4921369) [33]. The BrainSpan data are also available through the PsychENCODE Knowledge Portal: syn6136125 on Synapse.org (https://www.synapse.org/#!Synapse:syn6136125) [12, 34]. Scripts used in this manuscript can be found on Github [68].
