# Supplementary material for: Developmental dynamics of voltage-gated sodium channel isoform expression in the human and mouse brain
Source: Genome Med. 2021 Aug 23;13:135. doi: 10.1186/s13073-021-00949-0 (PMC8383430; doi:10.1186/s13073-021-00949-0)
Supplement: Supplementary file 1 — Additional file 1:. Supplementary Figures; Supplementary Figures 1 to 7 accompanying the main text. [file 13073_2021_949_MOESM1_ESM.pdf]

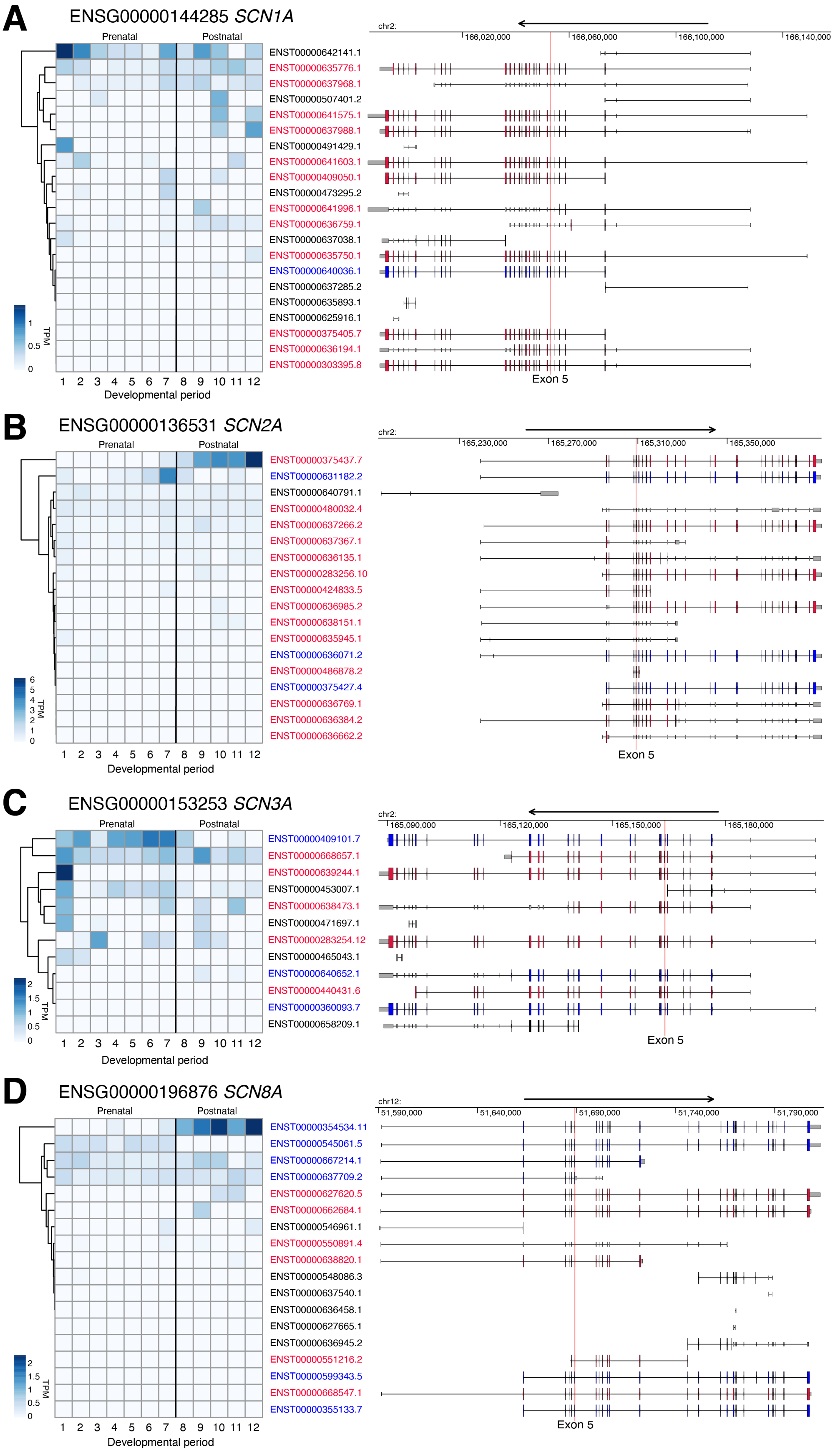
A
ENSG00000144285 SCN1A
Prenatal
Postnatal
TPM
1
0.5
0
1 2 3 4 5 6 7 8 9 10 11 12
Developmental period
ENST00000642141.1
ENST00000635776.1
ENST00000637968.1
ENST00000507401.2
ENST00000641575.1
ENST00000637988.1
ENST00000491429.1
ENST00000641603.1
ENST00000409050.1
ENST00000473295.2
ENST00000641996.1
ENST00000636759.1
ENST00000637038.1
ENST00000635750.1
ENST00000640036.1
ENST00000637285.2
ENST00000635893.1
ENST00000625916.1
ENST00000375405.7
ENST00000636194.1
ENST00000303395.8
chr2:
166,020,000
166,060,000
166,100,000
166,140,000
Exon 5
B
ENSG00000136531 SCN2A
Prenatal
Postnatal
TPM
6
5
4
3
2
1
0
1 2 3 4 5 6 7 8 9 10 11 12
Developmental period
ENST00000375437.7
ENST00000631182.2
ENST00000640791.1
ENST00000480032.4
ENST00000637266.2
ENST00000637367.1
ENST00000636135.1
ENST00000283256.10
ENST00000424833.5
ENST00000636985.2
ENST00000638151.1
ENST00000635945.1
ENST00000636071.2
ENST00000486878.2
ENST00000375427.4
ENST00000636769.1
ENST00000636384.2
ENST00000636662.2
chr2:
165,230,000
165,270,000
165,310,000
165,350,000
Exon 5
C
ENSG00000153253 SCN3A
Prenatal
Postnatal
TPM
2
1.5
1
0.5
0
1 2 3 4 5 6 7 8 9 10 11 12
Developmental period
ENST00000409101.7
ENST00000668657.1
ENST00000639244.1
ENST00000453007.1
ENST00000638473.1
ENST00000471697.1
ENST00000283254.12
ENST00000465043.1
ENST00000640652.1
ENST00000440431.6
ENST00000360093.7
ENST00000658209.1
chr2:
165,090,000
165,120,000
165,150,000
165,180,000
Exon 5
D
ENSG00000196876 SCN8A
Prenatal
Postnatal
TPM
2
1.5
1
0.5
0
1 2 3 4 5 6 7 8 9 10 11 12
Developmental period
ENST00000354534.11
ENST00000545061.5
ENST00000667214.1
ENST00000637709.2
ENST00000627620.5
ENST00000662684.1
ENST00000546961.1
ENST00000550891.4
ENST00000638820.1
ENST00000548086.3
ENST00000637540.1
ENST00000636458.1
ENST00000627665.1
ENST00000636945.2
ENST00000551216.2
ENST00000599343.5
ENST00000668547.1
ENST00000355133.7
chr12:
51,590,000
51,640,000
51,690,000
51,740,000
51,790,000
Exon 5

Figure S1. Isoform usage in voltage-gated sodium channels predicted by Salmon. A) For *SCN1A*, the estimated transcripts per million (TPM) values for each transcript in the human dorsolateral prefrontal cortex across twelve developmental periods are represented by the shade of blue in the heatmap, with transcripts with high estimated expression in darker shades. The names of transcripts that include exon 5A are shown in red, those that include exon 5N are shown in blue, while transcripts that do not include exon 5 are shown in black. Transcript definitions are from GENCODE v31 and genomic coordinates are shown in GRCh38. **B-D)** The representation in 'A' is repeated for *SCN2A*, *SCN3A*, and *SCN8A*. The Salmon postnatal results for *SCN8A* are inconsistent with the data for 5N and 5A (main manuscript).

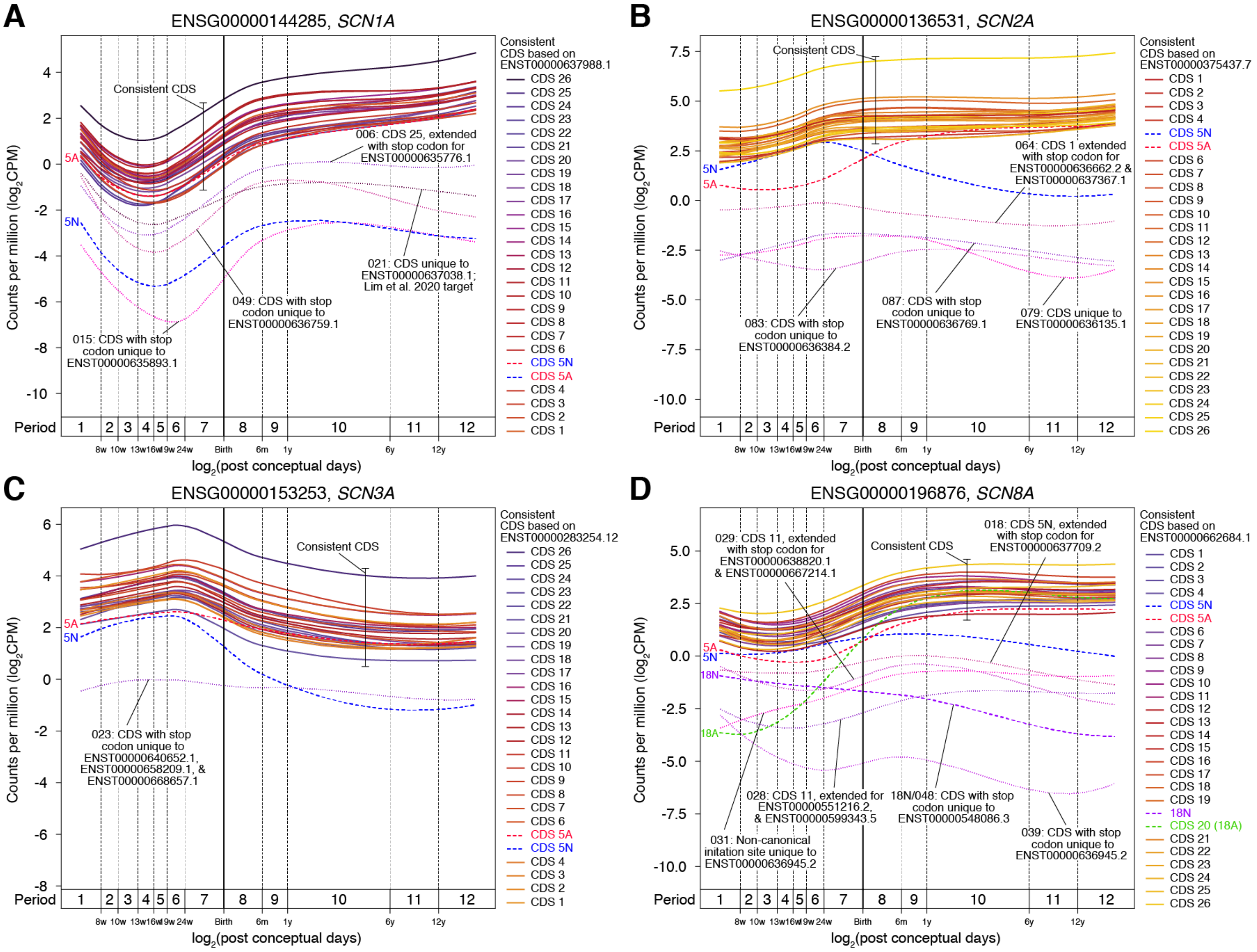

Figure S2. Expression of all protein-coding exons in voltage-gated sodium channels in the human cortex across development. A) The Loess smoothed average expression of each protein-coding exon in *SCN1A* is shown across development. Constitutive protein-coding exons (CDS) are labelled together, and their corresponding color shown in the legend. Variable CDS, that are unique to a small number of transcripts, are labeled along with their DEXSeq exon code (Additional file 3: Table S2). **B-D)** Panel 'A' is repeated for *SCN2A*, *SCN3A*, and *SCN8A*.

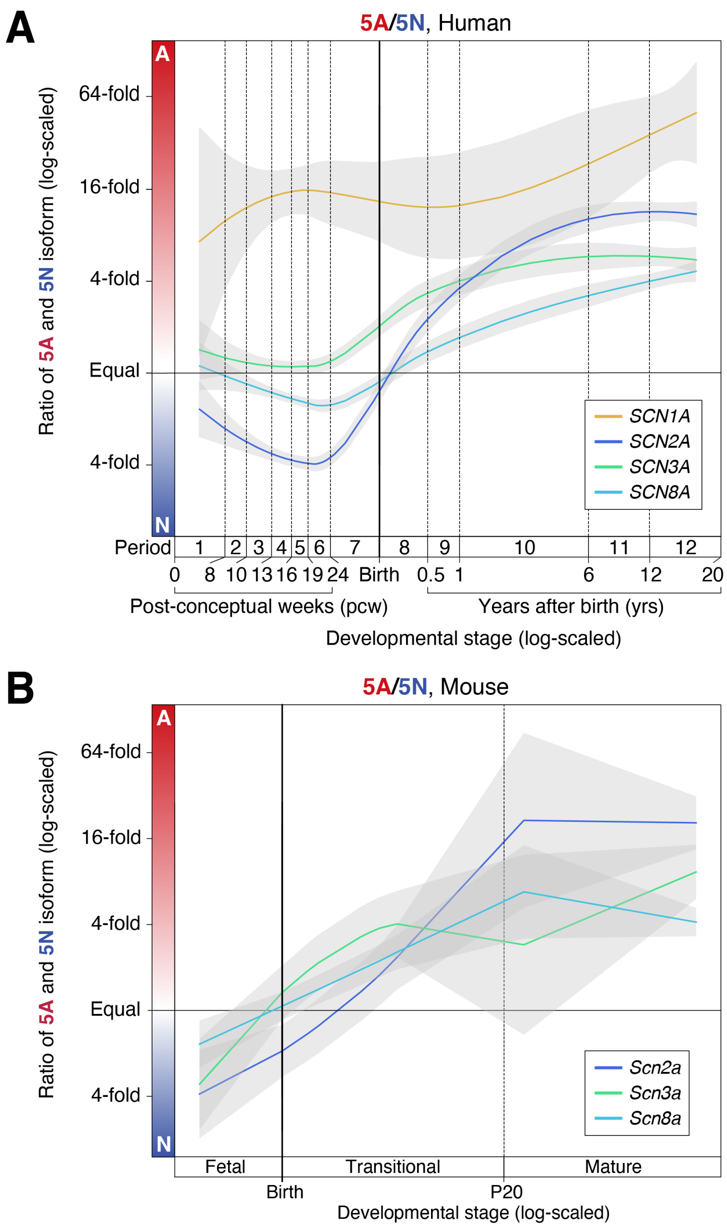

Figure S3. Ratio of 5A and 5N expression in the human and mouse cortex across development. A) The ratio of 5A and 5N expression for each sodium channel is shown across development for 176 BrainVar human cortex (DLPFC) samples across development; the colored lines show the Loess smoothed average and 95% confidence interval (shaded region). **B)** Panel A is repeated for 58 mouse cortex samples across development.

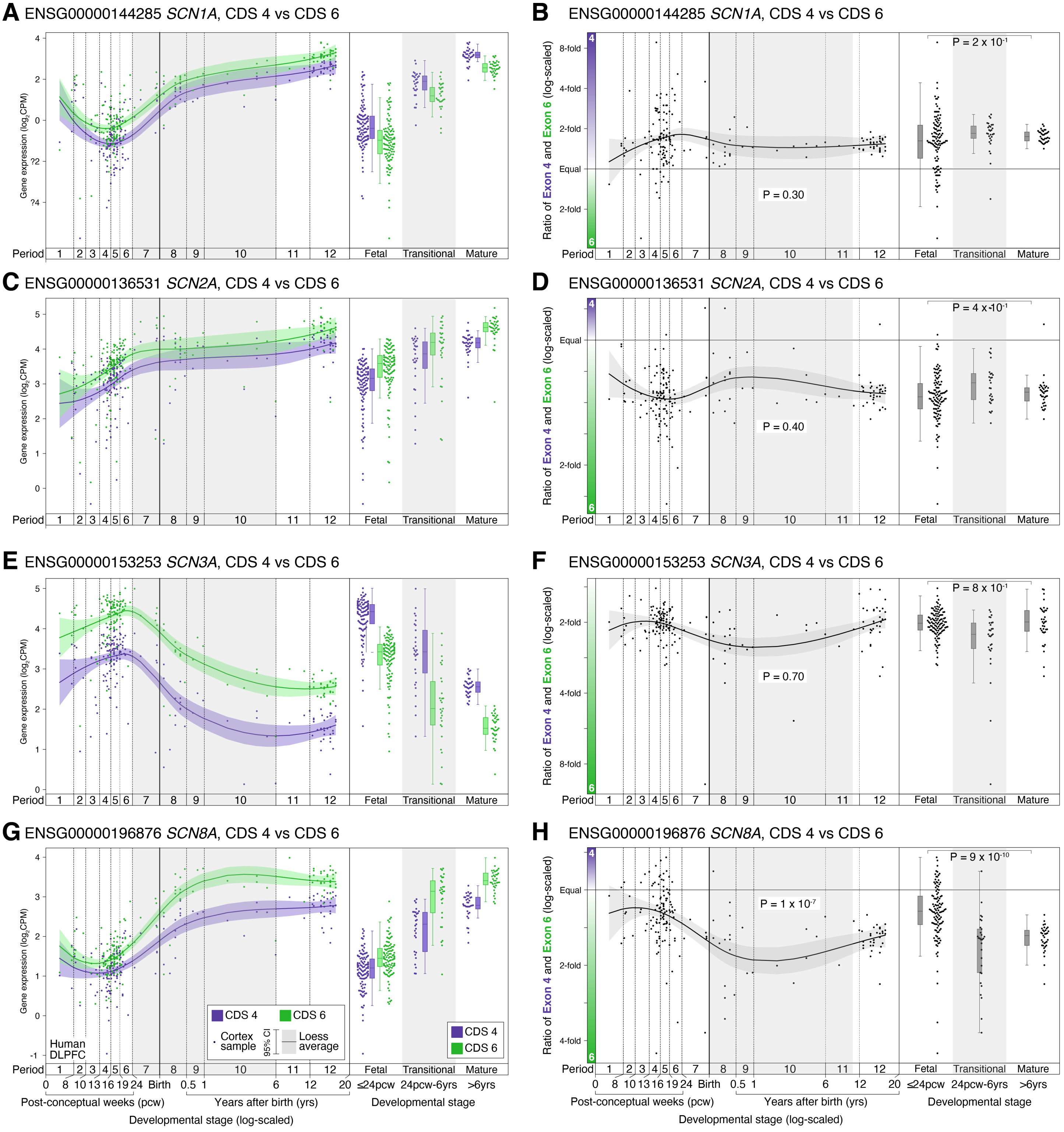

Figure S4. Expression of CDS 4 and CDS 6 in the human cortex across development. A) The expression of CDS 4 (purple) and CDS 6 (green) in *SCN1A* is shown for 176 BrainVar human dorsolateral prefrontal cortex samples across development (points). On the left, the colored line shows the Loess smoothed average and 95% confidence interval (shaded region). On the right, boxplots show the median and interquartile range for the same data, binned into fetal, transitional, and mature developmental stages. **B)** The ratio of CDS 4 and CDS 6 expression from panel 'A' is shown across development (left) and in three developmental stages (right). **C-H)** Panels A and B are repeated for the genes *SCN2A*, *SCN3A*, *SCN8A*. CPM: Counts per million. Statistical tests: B, D, F, H) Left panel, linear regression of $\log_2$(4:6 ratio) and $\log_2$(post-conceptual days). Right panel, two-tailed Wilcoxon test of $\log_2$(4:6 ratio) values between fetal and mature groups.

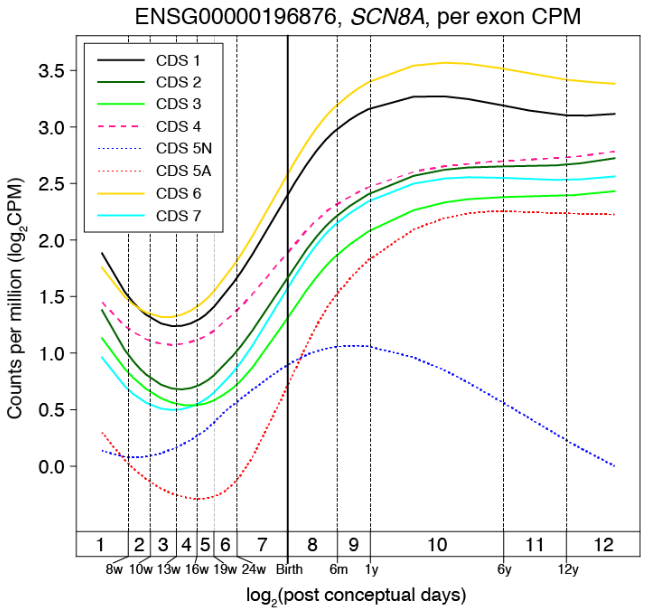

Figure S5. Expression of *SCN8A* constitutive CDS 1-7 in the human cortex. The Loess smoothed average expression in the dorsolateral prefrontal cortex is shown across human development. CDS 4 is highlighted in red with a dashed line. These data are a subset of Additional file 1: Fig. S2D.

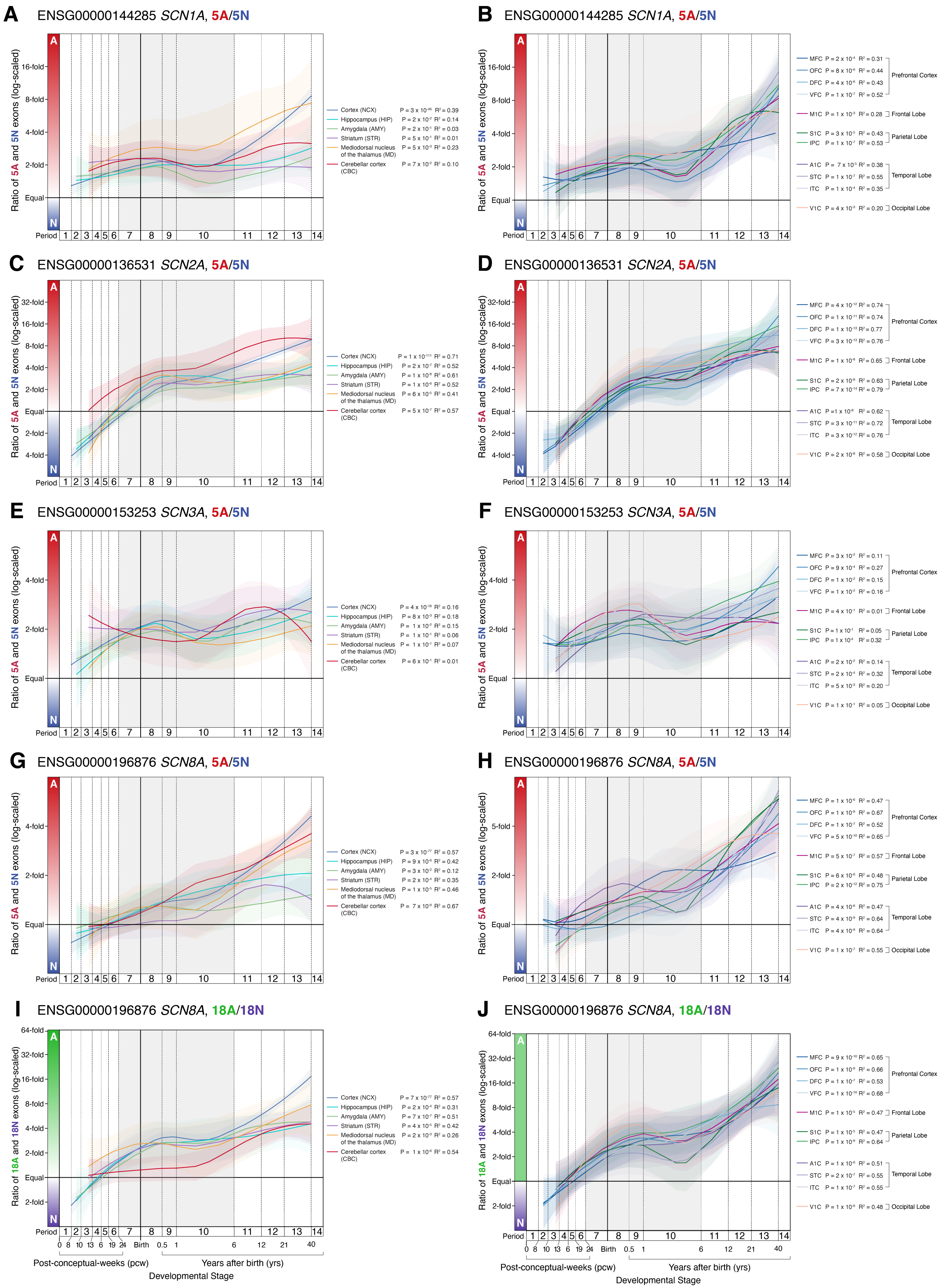
A ENSG00000144285 SCN1A, 5A/5N
B ENSG00000144285 SCN1A, 5A/5N
C ENSG00000136531 SCN2A, 5A/5N
D ENSG00000136531 SCN2A, 5A/5N
E ENSG00000153253 SCN3A, 5A/5N
F ENSG00000153253 SCN3A, 5A/5N
G ENSG00000196876 SCN8A, 5A/5N
H ENSG00000196876 SCN8A, 5A/5N
I ENSG00000196876 SCN8A, 18A/18N
J ENSG00000196876 SCN8A, 18A/18N
Ratio of 5A and 5N exons (log-scaled)
Ratio of 18A and 18N exons (log-scaled)
Equal
Period
Cortex (NCX)
Hippocampus (HIP)
Amygdala (AMY)
Striatum (STR)
Mediodorsal nucleus of the thalamus (MD)
Cerebellar cortex (CBC)
MFC
OFC
DFC
VFC
Prefrontal Cortex
M1C
Frontal Lobe
S1C
IPC
Parietal Lobe
A1C
STC
ITC
Temporal Lobe
V1C
Occipital Lobe
Birth
Post-conceptual-weeks (pcw)
Years after birth (yrs)
Developmental Stage

Figure S6. Expression of CDS 5A and 5N in 16 human brain regions across development. A) The ratio of CDS 5A and CDS 5N expression is shown across development for *SCN1A* in 607 BrainSpan human *postmortem* brain samples from 41 individual brains in six brain regions (indicated by color). The colored line shows the Loess smoothed average and 95% confidence interval (shaded region). **B)** The cortex samples from 'A' are split into 11 cortical regions (indicated by color). C-H) The analyses in 'A' and 'B' are repeated for *SCN2A*, *SCN3A*, and *SCN8A*. Abbreviations: OFC: orbital prefrontal cortex; DFC: dorsolateral prefrontal cortex; VFC: ventrolateral prefrontal cortex; MFC: medial prefrontal cortex; M1C: primary motor (M1) cortex; S1C: primary somatosensory (S1) cortex; IPC: posterior inferior parietal cortex; A1C: primary auditory (A1) cortex; STC: superior temporal cortex; ITC: inferior temporal cortex; V1C: primary visual (V1) cortex. Statistical tests: A-H) Linear regression of $\log_2$(5A:5N ratio + 0.1) and $\log_2$(post-conceptual days).

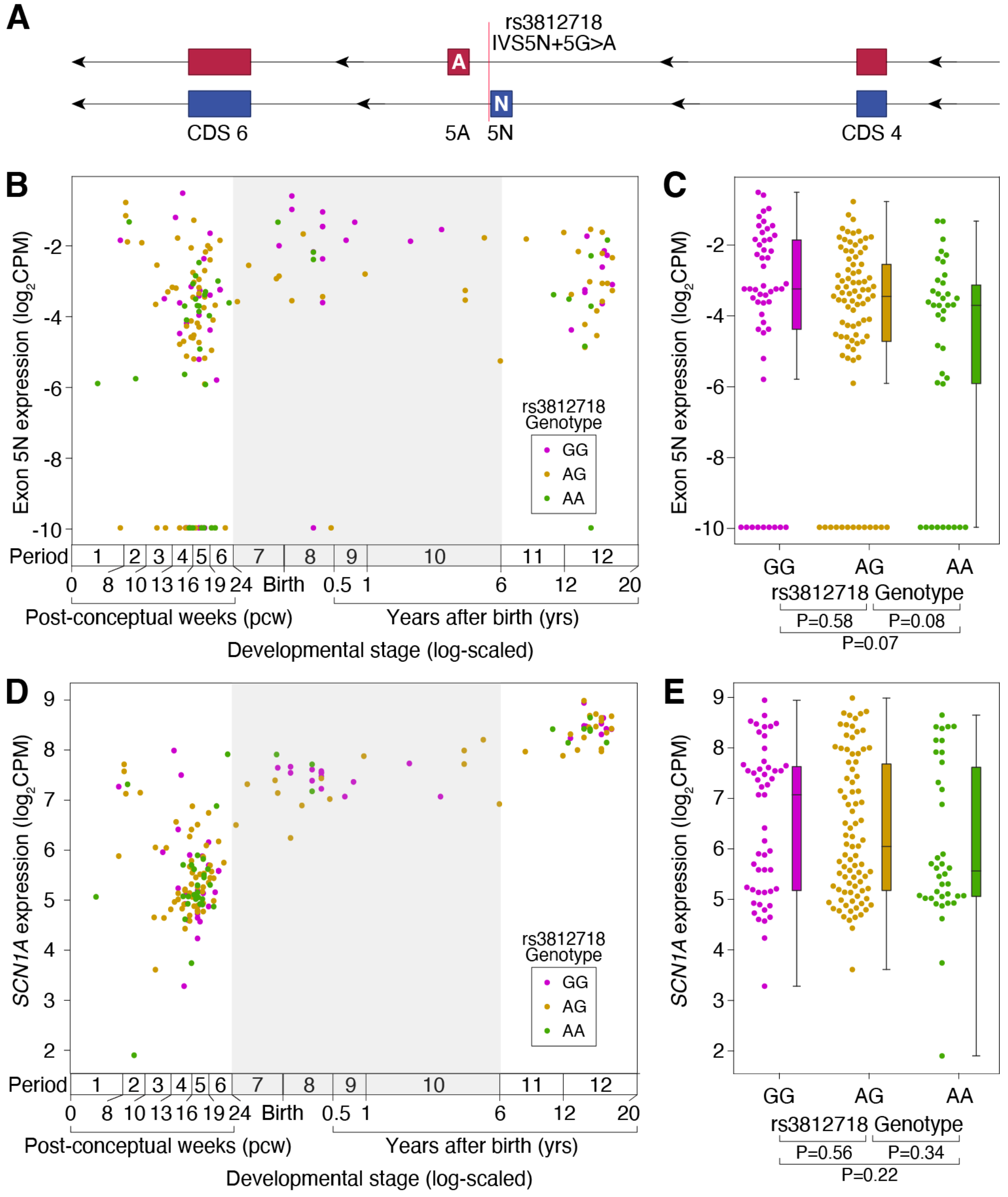

Figure S7. 5N expression by rs3812718 genotype in the developing human cortex. A) rs3812718 (chr2:166,053,034 C>T, GRCh38) is a common single nucleotide polymorphism (SNP) previously associated with differential 5N inclusion in the human temporal cortex.[21,22] The SNP is five nucleotides upstream of the 5N donor splice site. **B)** Normalized 5N expression by rs3812718 genotype in 176 *postmortem* human dorsolateral prefrontal cortex samples across cortical development. Expression values are $\log_2$-scaled counts per million ($\log_2$CPM) with a value of -10 representing no reads detected (31 samples). **C)** Normalized 5N expression ($\log_2$CPM) is shown for the same samples as in 'B', binned by genotype. **D)** Panel 'B' is repeated for *SCN1A* gene-wide expression. **E)** Panel 'C' is repeated for *SCN1A* gene-wide expression. Statistical tests: C, D) Two-tailed Wilcoxon test.
