# Supplementary material for: Developmental dynamics of voltage-gated sodium channel isoform expression in the human and mouse brain
Source: Genome Med. 2021 Aug 23;13:135. doi: 10.1186/s13073-021-00949-0 (PMC8383430; doi:10.1186/s13073-021-00949-0)
Supplement: Supplementary file 4 — Additional file 4:. Supplementary materials [file 13073_2021_949_MOESM4_ESM.docx]

**Supplementary Materials**

**Data Acquisition**

STAR aligned BAMs from the 176 BrainVar bulk-tissue RNA-seq samples were obtained from the PsychENCODE Knowledge Portal: syn21557948 on Synapse.org (https://www.synapse.

org/#!Synapse:syn4921369), as described previously [33]. The BrainSpan bulk-tissue RNA-seq BAMs were also obtained from the PsychENCODE Knowledge Portal: syn6136125 [12,34]. For the mouse data, FASTQ files for the mouse data for development periods before P28 were generated as controls for ongoing experiments. FASTQ files for samples at or after P28 were obtained from NCBI’s Sequence Read Archive [35] (Additional file 2: Table S1). Sample ages for the BrainVar samples, expressed as post-conceptual days were obtained from the supplementary materials [33].

**RNA-seq alignment and exon-level read count quantification**

For the mouse data, RNA-seq short reads were aligned with the STAR aligner to the *Mus musculus* reference GRCm38.p6 to generate BAM files equivalent to the BrainVar data. For both the BrainVar and mouse datasets, exon level read counts for the genes *SCN1A*/*Scn1a* (ENSG00000144285.20/ENSMUSG00000064329.13), *SCN2A*/*Scn2a* (ENSG00000136531.16/ENSMUSG00000075318.13), *SCN3A*/*Scn3a* (ENSG00000153253.18/ENSMUSG00000057182.15), and *SCN8A*/*Scn8a* (ENSG00000196876.16/ENSMUSG00000023033.14) were calculated using DEXSeq [37], annotating to GENCODE v31 of the GRCh38.p12 human reference genome in humans and GENCODE vM25 of the GRCm38.p6 mouse reference genome in mice. GENCODE GTF files were converted to GFF files using the DEXSeq package script “dexseq_prepare_annotation.py.” Counts were acquired from the ‘dexseq_count.py script’. BrainVar samples were processed as paired-end and reverse stranded (-p yes -s reverse), BrainSpan samples were processed as single-end and unstranded (-p no -s no), and mouse samples were processed with a combination of paired- and single-endedness and stranded and unstrandedness (Additional file 2: Table S1). Raw read counts were converted into counts per million (CPM) [38].

**Transcript level quantification**

BAMs generated for the BrainVar dataset were converted to paired-end FASTQ files using bedtools v.2.29.2 bamtofastq command [69]. Transcripts were quantified with “salmon quant -i transcript_idx -l A -1 sample_r1.fastq.gz -2 sample_r2.fastq.gz -o output --validateMappings --gcBias –dumpEq” with version 1.3.0 of the software [70]. The transcript index was generated using all GRCh38 transcript sequences from GENCODE v31.

**Exon junction quantification**

Short reads from the BrainVar dataset were aligned to the human reference genome GRCh38.p12 using OLego v.1.1.5 [40]. The resulting BAMs were converted into junction files and run through Leafcutter’s suite of tools for quantifying RNA-splicing variation [41]. Introns found in the junction files were clustered together with the default setting of 50 reads per cluster and a maximum intron length of 500 kb, creating a matrix describing the read count per sample at a particular intron cluster site using the default parameters to the “leafctter_cluster.py” script. The file used to define exons was derived from the GENCODE v31 GTF for GRCh38 and is hosted in the Leafcutter git repo [68]. Differential splicing analysis was run based on whether samples were identified as prenatal or postnatal using the default parameters to the “leafcutter_ds.R” script. The effect sizes outputted from the differential splicing analysis were processed with Leafcutter’s “Leafviz” visualization tool to annotate introns.

**References**

69. Quinlan AR, Hall IM. BEDTools: a flexible suite of utilities for comparing genomic features. Bioinformatics. 2010;26(6):841–2. <https://doi.org/10.1093/bioinformatics/btq033>.

70. Patro R, Duggal G, Love MI, Irizarry RA, Kingsford C. Salmon provides fast and bias-aware quantification of transcript expression. Nat Methods. 2017;14(4):417–9. <https://doi.org/10.1038/nmeth.4197>.
